# Supplementary material for: Impact of tides and sea-level on deep-sea Arctic methane emissions
Source: Nat Commun. 2020 Oct 9;11:5087. doi: 10.1038/s41467-020-18899-3 (PMC7547717; doi:10.1038/s41467-020-18899-3)
Supplement: Supplementary file 1 — Supplementary Information [file 41467_2020_18899_MOESM1_ESM.pdf]

Supplementray material for

Impact of tides and sea-level on deep-sea Arctic methane emissions

Nabil Sultan[1*], Andreia Plaza-Faverola[2], Sunil Vadakkepuliyambatta[2], Stefan Buenz[2], Jochen Knies[2,3]

[1] Ifremer, Département REM, Unité des Géosciences Marines, F-29280 Plouzané, France

N. S. nabil.sultan@ifremer.fr

[2] Centre for Arctic Gas Hydrate, Environment and Climate, Department of Geosciences, UiT - The Arctic University of Norway in Tromsø, Norway

A. P.-F. andreia.a.faverola@uit.no

S. V. sunil.vadakkepuliyambatta@uit.no

S. B. stefan.buenz@uit.no

[3] Geological Survey of Norway, NO-7491, Trondheim, Norway

J. K. Jochen.Knies@ngu.no

*** Corresponding author**

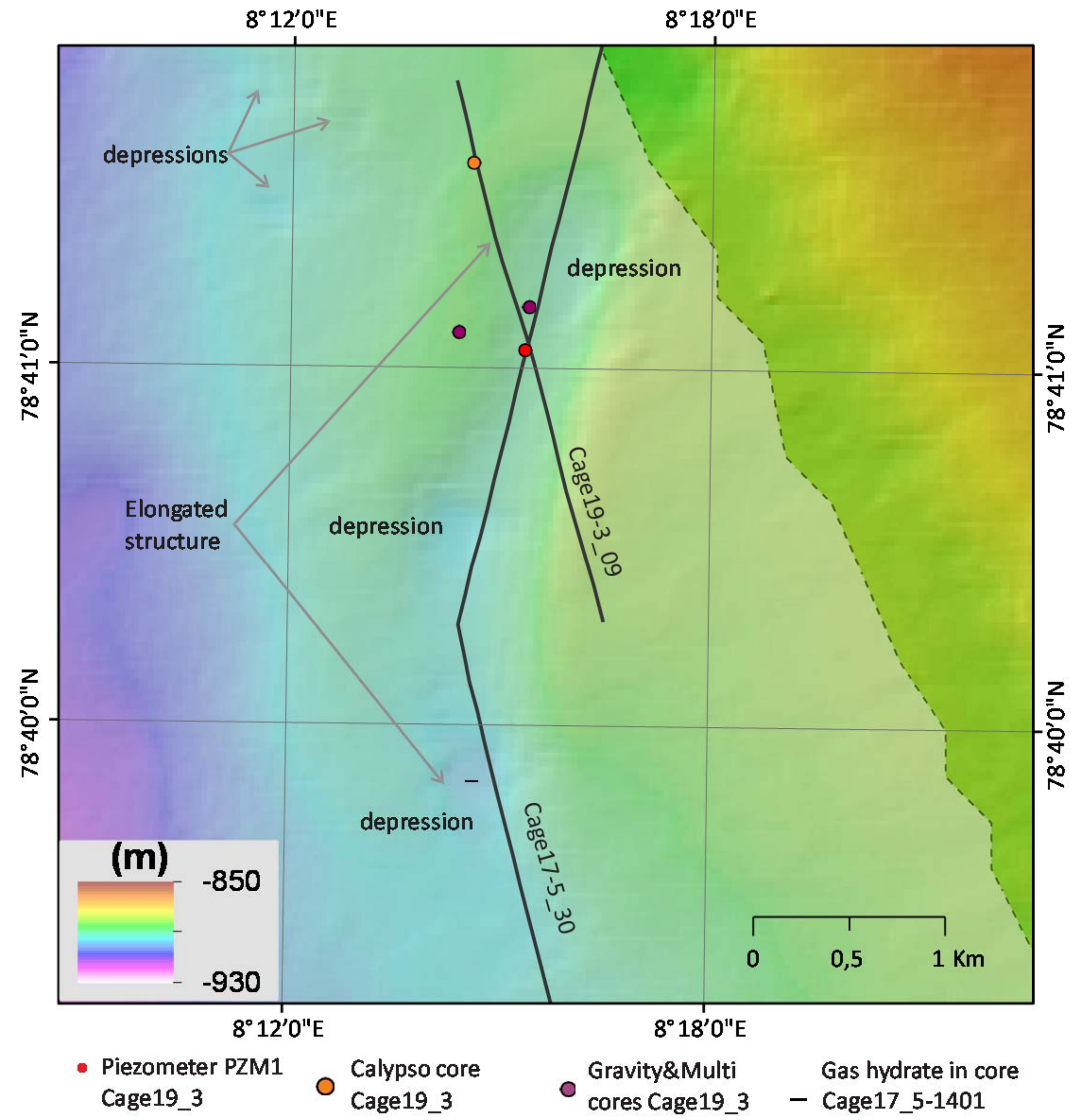

Supplementary figure 1: Bathymetry acquired with Kongsberg Simrad EM 302 multi-beam echo sounder mounted on R/V Helmer Hanssen. Piezometer site 1 (PZM1) is located ca. 1.5 km north from a gravity core (CAGE17-5-1401) where gas hydrate nodules were sampled at 1.5 mbsf (Figure 2 – Cage17-5 cruise report https://cage.uit.no/cruise/geomorphic-mapping-seismic-stratigraphy-cruise/). Both gravity core with hydrates and the piezometer site are located along an elongated depression that contains smaller depressions of about 300 to 400 meter width. The shaded area marks the gas hydrate stability zone based on the presence of gas-hydrate related bottom simulating reflections in seismic profiles[1].

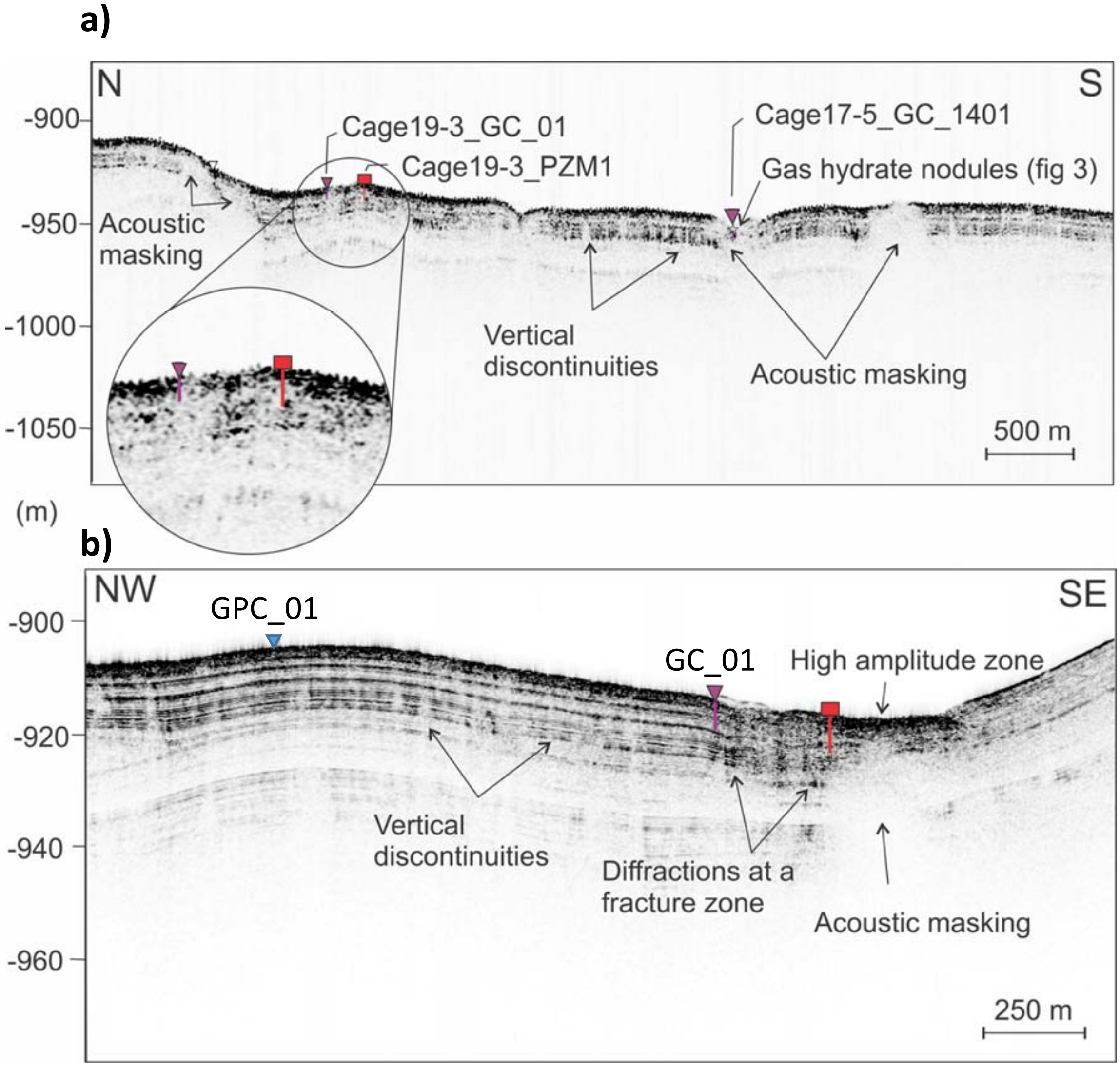

Supplementary figure 2: Sub-bottom profiles along (Cage17-5_30 in a) and across (Cage19-3_09 in b) the elongated structure with associated focused vertical fluid migration pathways. Depth ranges correspond to a velocity of 1500 m/s. The elongated structure extends for ~ 3 km in a north south direction (Supplementary figure 1) and contains domes and depressions with associated transparent facies that are typically associated with gas chimneys or pipes. The masking effect indicates the presence of material that attenuates the acoustic energy. Gas in the sediment has seismic attenuation properties whilst gas hydrate accumulations appear to enhance the energy[2]. PZM1 is located within one of the focused vertical fluid migration features characterized by a highly fractured zone at the flank of a depression, a transparent facies indicating the main focus of fluid migration toward the seafloor and a ca. 2-3 m thick high amplitude zone near the seafloor that possibly contains gas hydrate nodules and/or methane-derived authigenic carbonates material (i.e., the product of past methane seepage). In 2017 one of the zones with transparent facies was sampled using a gravity core and gas hydrate nodules (Supplementary figure 3) were retrieved between 0.7-1.2 mbsf (Cage17-5_1401 gravity core).

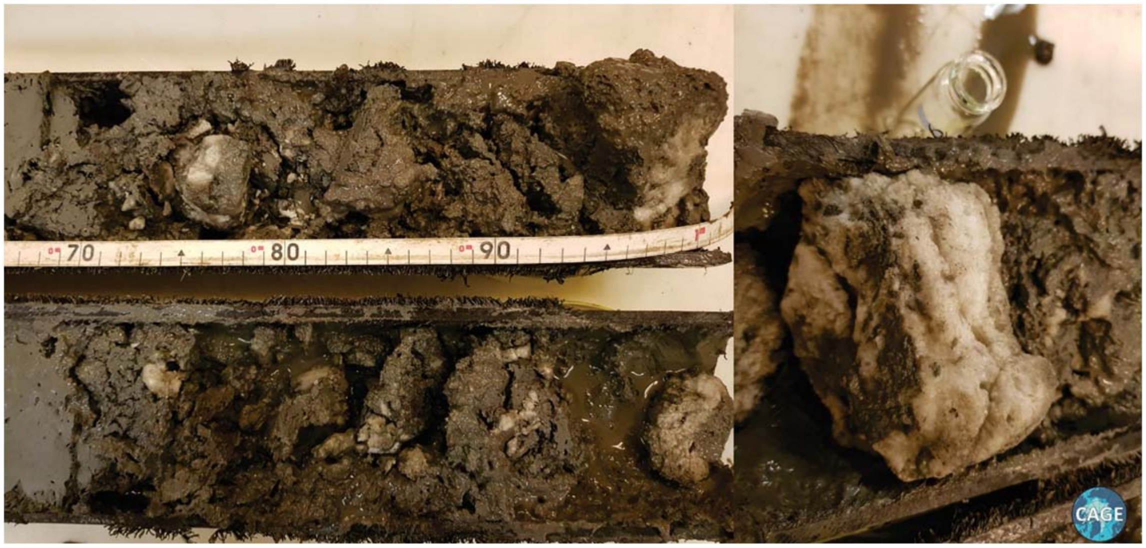

Supplementary figure 3: Pictures of gas hydrate nodules retrieved within 0.7-1.2 mbsf on gravity core CAGE17-5_1401 (Cruise report - https://cage.uit.no/cruise/geomorphic-mapping-seismic-stratigraphy-cruise/). Photo/Image courtesy of Karin Andreassen (CAGE-UiT The Arctic University of Norway).

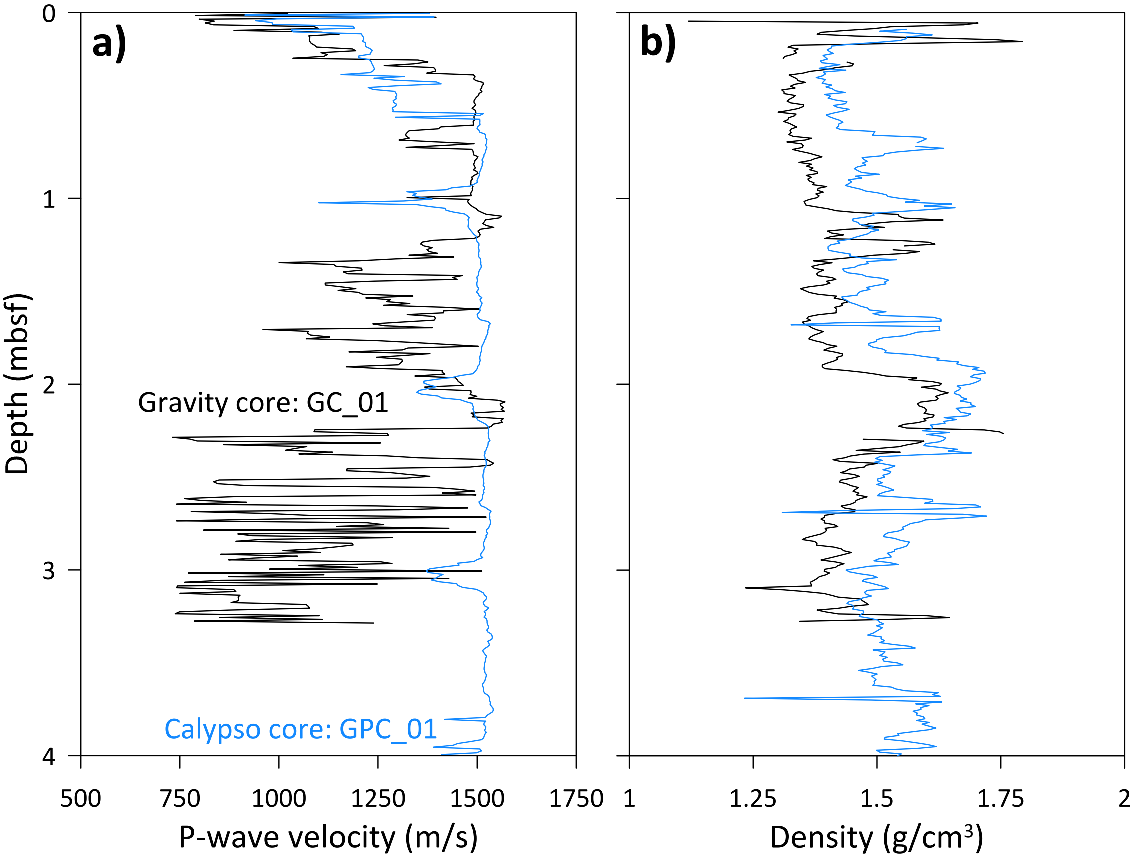

Supplementary figure 4: P-wave velocity (a) and density (b) profiles from gravity GC_01 and Calypso GPC_01 cores. Location of cores are shown in supplementary figures 1 & 2. Low P wave velocities measured from GC_01 possibly indicate the presence of in-situ dissolved gas below 2.1 mbsf.

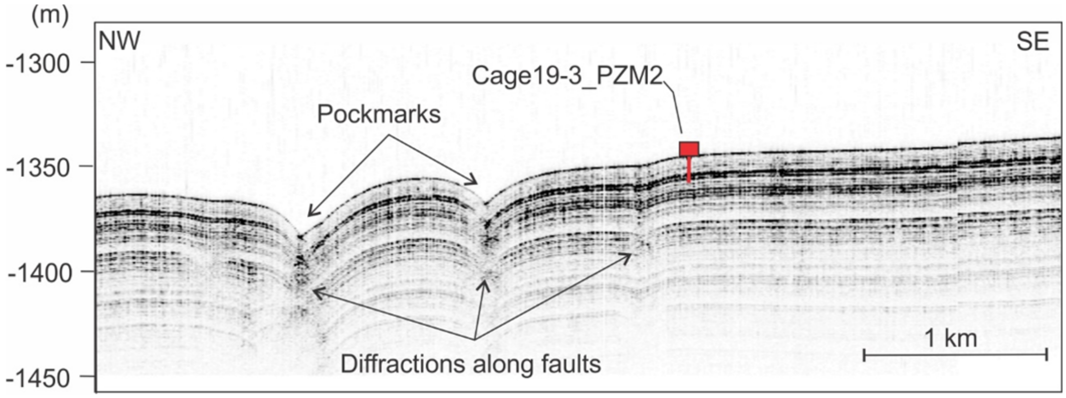

Supplementary figure 5: Sub-bottom profile (CAGE19-3_10) along the western Vestnesa Ridge segment showing continuous reflections and homogenous facies in the surrounding of piezometer PZM2 site. See figure 1 in the main text for location.

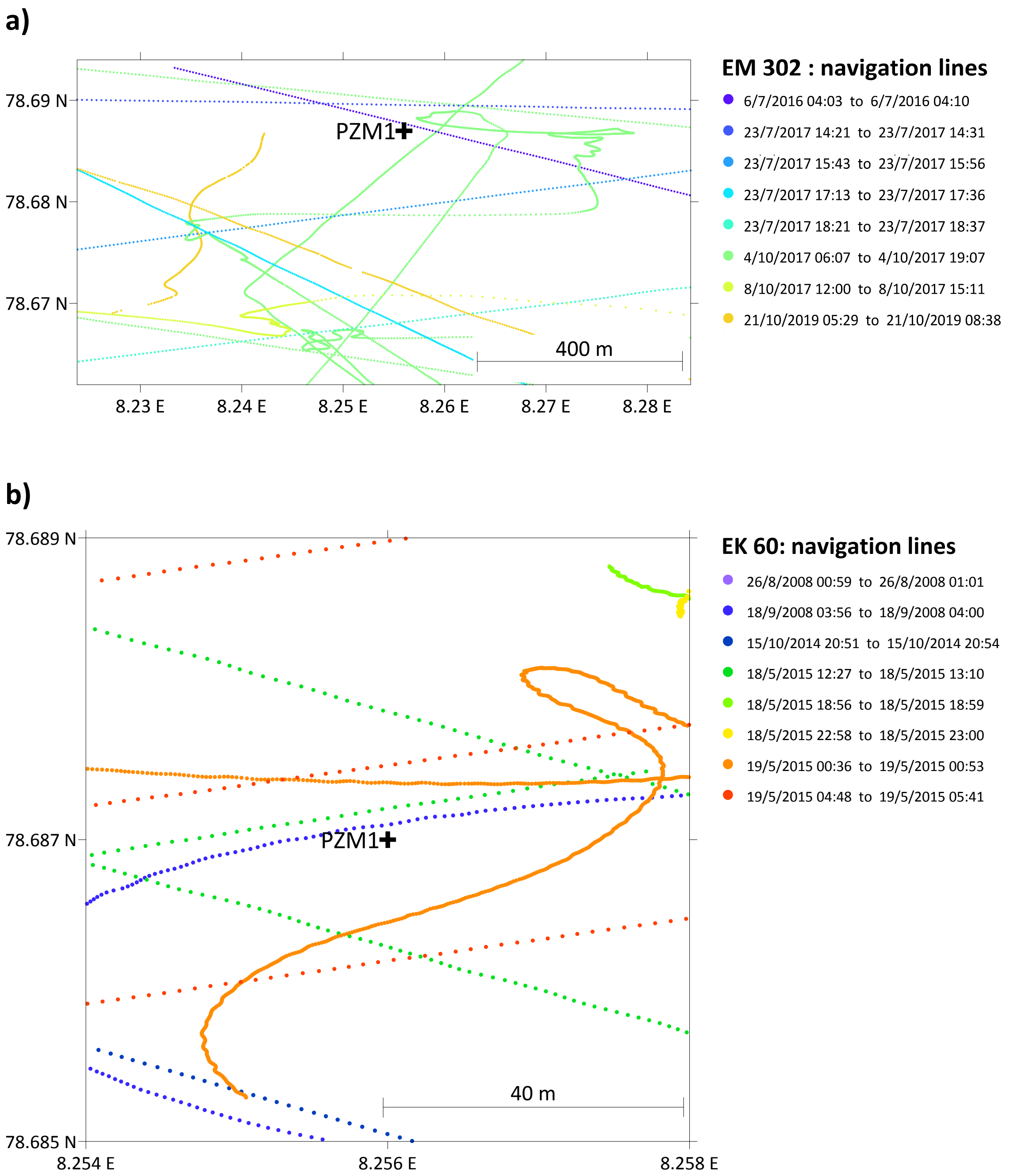

Supplementary figure 6. Navigation data corresponding to available a) EM302 (multi-beam sonar) and b) EK60 (single beam sonar) hydro-acoustic profiles from site PZM1. Colors correspond to acquisition dates. No gas seep was detected during the EM302 and EK60 sonar data acquisition.

EM 302

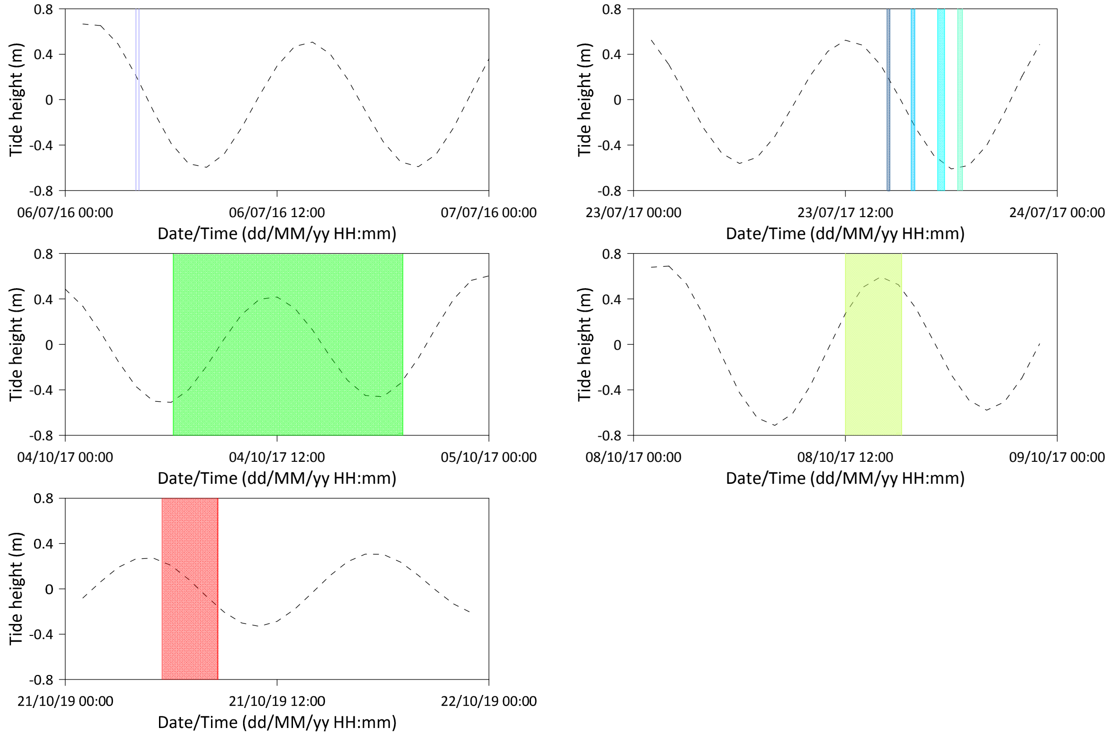

EK 60

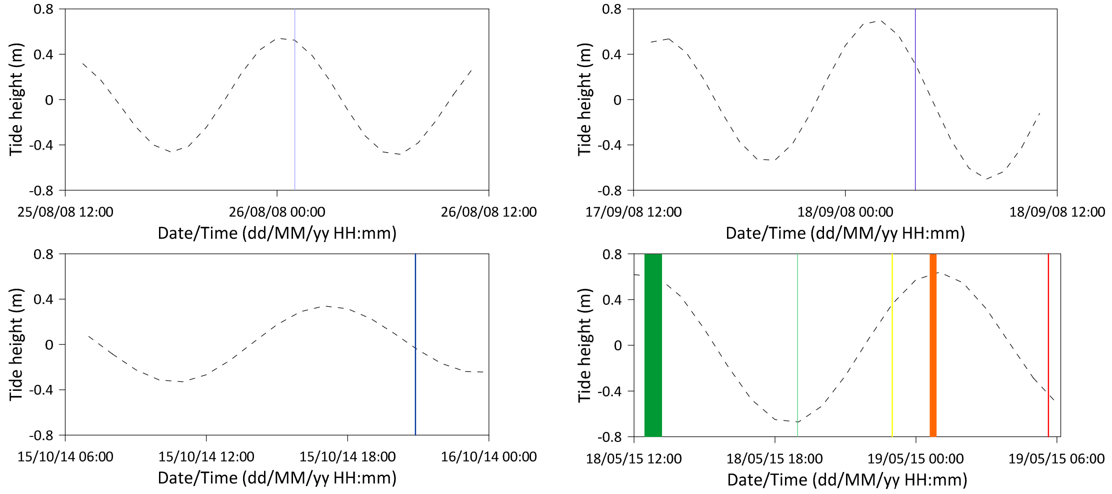

Supplementary figure 7. Tidal height derived at the piezometer location from the TPXO 9.0 global tidal model[3,4] during hydro-acoustic surveys. Color vertical lines refer to the acquisition date within 1500 m distance for EM302 and 50 m distance for EK 60 from PZM1 (for position see Supplementary figure 1). Dashed zones indicate multi-lines hydro-acoustic-surveys.

a)

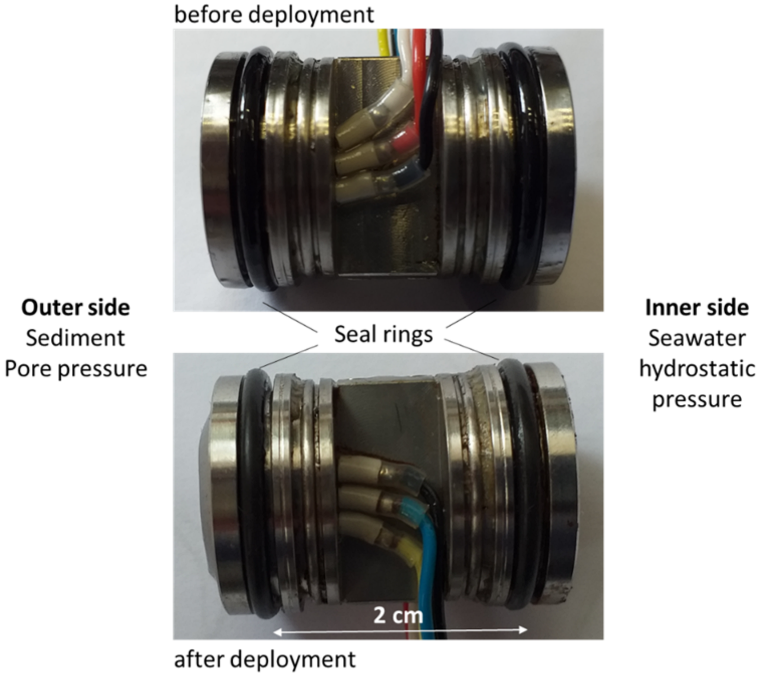

b) Outer side (before and after deployment) : pressure lower than hydrostatic

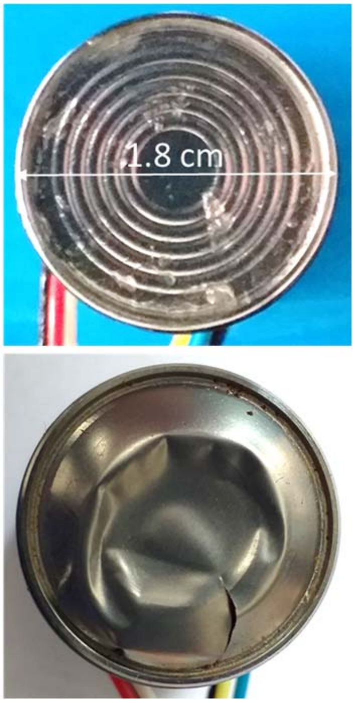

c) Inner side (before and after deployment): hydrostatic pressure

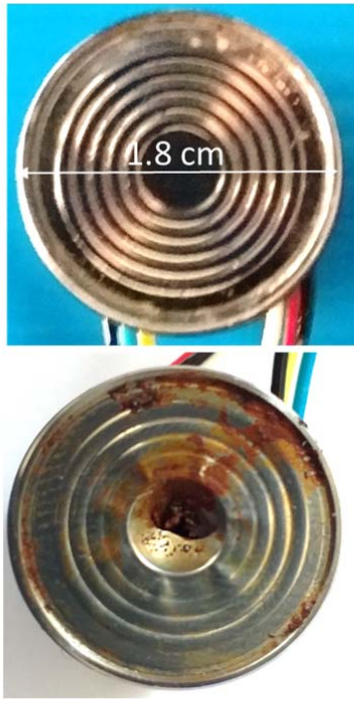

Supplementary figure 8. a) Differential pore pressure sensor mounted horizontally in the piezometer with an outer side subjected to the sediment pore pressure and inner side exposed to the hydrostatic pressure. Supposed high gas emissions encountered during a deployment at a third planned site along the Vestnesa Ridge offshore Svalbard damaged the pore pressure sensor after one hour of recording. In comparison to the initial shape of both faces, the curved shape of the outer side (b) and the collapse of the inner side (c) suggest the occurrence of high negative differential pore pressure prior to failure (inner pressure higher than the outer pressure).

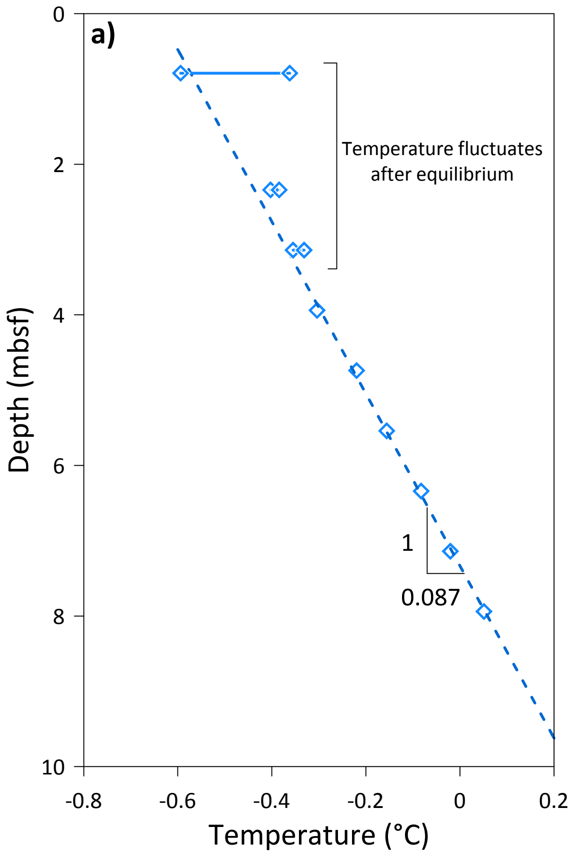

Supplementary figure 9. Thermal gradients derived from a) PZM1 and b) PZM2 data.

Supplementary References

1 Minshull, T. A. *et al.* Hydrate occurrence in Europe: A review of available evidence. *Marine and Petroleum Geology* **111**, 735-764, doi:10.1016/j.marpetgeo.2019.08.014 (2020).

2 Singhroha, S., Bunz, S., Plaza-Faverola, A. & Chand, S. Gas hydrate and free gas detection using seismic quality factor estimates from high-resolution P-cable 3D seismic data. *Interpretation-a Journal of Subsurface Characterization* **4**, SA39-SA54, doi:10.1190/int-2015-0023.1 (2016).

3 Egbert, G. D. & Erofeeva, S. Y. Efficient inverse Modeling of barotropic ocean tides. *Journal of Atmospheric and Oceanic Technology* **19**, 183-204, doi:10.1175/1520-0426(2002)019<0183:eimobo>2.0.co;2 (2002).

4 Egbert, G. D., Bennett, A. F. & Foreman, M. G. G. TOPEX/POSEIDON TIDES ESTIMATED USING A GLOBAL INVERSE MODEL. *Journal of Geophysical Research-Oceans* **99**, 24821-24852, doi:10.1029/94jc01894 (1994).
